# Optimization of beam angles for intensity modulated radiation therapy treatment planning using genetic algorithm on a distributed computing platform

**DOI:** 10.4103/0971-6203.54845

**Published:** 2009

**Authors:** Daryl P. Nazareth, Stephen Brunner, Matthew D. Jones, Harish K. Malhotra, Mohammad Bakhtiari

**Affiliations:** Department of Radiation Medicine, Roswell Park Cancer Institute, Elm & Carlton Sts, Buffalo NY 14263, USA

**Keywords:** Intensity modulated radiation therapy, distributed computing, genetic algorithm, optimization

## Abstract

Planning intensity modulated radiation therapy (IMRT) treatment involves selection of several angle parameters as well as specification of structures and constraints employed in the optimization process. Including these parameters in the combinatorial search space vastly increases the computational burden, and therefore the parameter selection is normally performed manually by a clinician, based on clinical experience. We have investigated the use of a genetic algorithm (GA) and distributed-computing platform to optimize the gantry angle parameters and provide insight into additional structures, which may be necessary, in the dose optimization process to produce optimal IMRT treatment plans. For an IMRT prostate patient, we produced the first generation of 40 samples, each of five gantry angles, by selecting from a uniform random distribution, subject to certain adjacency and opposition constraints. Dose optimization was performed by distributing the 40-plan workload over several machines running a commercial treatment planning system. A score was assigned to each resulting plan, based on how well it satisfied clinically-relevant constraints. The second generation of 40 samples was produced by combining the highest-scoring samples using techniques of crossover and mutation. The process was repeated until the sixth generation, and the results compared with a clinical (equally-spaced) gantry angle configuration. In the sixth generation, 34 of the 40 GA samples achieved better scores than the clinical plan, with the best plan showing an improvement of 84%. Moreover, the resulting configuration of beam angles tended to cluster toward the patient's sides, indicating where the inclusion of additional structures in the dose optimization process may avoid dose hot spots. Additional parameter selection in IMRT leads to a large-scale computational problem. We have demonstrated that the GA combined with a distributed-computing platform can be applied to optimize gantry angle selection within a reasonable amount of time.

## Introduction

Intensity Modulated Radiation Therapy (IMRT) is a complex technology designed to deliver precisely-modulated and conformal radiation dose to a target. IMRT is employed clinically for several treatment sites, including the prostate, the head and neck region, and the brain.

IMRT planning requires the solution to the beam-angle selection (BAS) problem,[1–8] which involves the selection of 5-10 angles from 360 possible gantry angles, subject to certain spacing and opposition constraints. This type of optimization problem is termed an integer programming (IP) problem, and involves a large number of binary variables. For example, in selecting 5-10 angles out of a set of 360, there are approximately 9 × 10^19^ possible candidates. In addition, in many clinics, the rotation angles of the treatment couch are also considered, further increasing the number of variables and possible solutions. Since no commercial software package exists which solves the BAS problem, clinicians generally select the gantry and couch angles manually based on clinical experience.

A second IMRT sub-problem is that of dose optimization (DO).[9–19] This problem involves optimizing beamlet weights in order to define an intensity map for each radiation field. The constraints involved are usually expressed as dose-volume histogram (DVH) constraints on targets and organs at risk. As noted above, no existing commercial technique attempts to solve the BAS and DO problems simultaneously. Other investigators have employed genetic algorithm approaches[20,21] to the BAS problem, but have not coupled it with a standard commercial dose optimization routine. As described below, we use the GA as an “outer loop” of our complete optimization technique.

The major impediment to solving the BAS, simultaneously with standard IMRT optimization, is the prohibitive size of the solution search space. However, the advent of high-performance computing and computer clusters has now made such attempts feasible. Our work involves the development of distributed-computing tools to solve simultaneously the BAS and DO problems. These tools would represent significant improvement in current clinical IMRT treatment-planning implementation.

## Methods

### B1. Genetic Algorithm

Genetic algorithm[22–27] (GA) is an adaptive heuristic search technique based on the biological principles of evolution and natural selection. It employs an initial random population which effectively samples the search space globally, along with a propagation technique to concentrate the search effort in promising regions. In this work, we will employ the GA to guide the search for optimal beam angles while simultaneously generating IMRT treatment plans.

The GA approach includes four steps: (1) producing the first generation of beam-angle sample sets, (2) determining the “most fit” samples by means of a scoring function (3) combining the most promising samples using a crossover technique (4) applying a mutation scheme to a selection of the new generation of samples.

The search space consists of all candidate sets of gantry beam angles, subject to the following constraints: angles which differ by less than 30° are not permitted, and angles within 30° of opposition are not permitted. These are typical constraints employed in clinical IMRT treatment planning. The number of beams will be selected by the user, based on clinical considerations, although further development in this work would permit this parameter to be included in the search. As an example, we consider the treatment plan for a prostate patient in which the number of beams, *N* is equal to five. Producing the initial population of *k* samples is accomplished by random number generation.

For each of the *k* samples, an IMRT treatment plan is generated using the Eclipse software (Varian Medical Systems, Palo Alto, CA), with clinically-relevant DVH constraints. Currently, each plan is generated manually, as in standard clinical treatment planning; an automated method using a distributed-computing approach is described in the ‘’Discussion and Conclusions” section. Each plan then receives a score according to how well it satisfies the constraints; this enables the samples to be ranked. The best sample is *cloned* or copied, unchanged, to the next generation. The remaining *k-1* positions of the next generation are assigned to *offspring* of the best samples using the following method. For each pair of samples in the current generation, the average score is calculated. The best *k-1* pairs are placed into the *mating pool*. For each pair in this pool, a random binary *crossover mask* is generated and used to determine the beams angles of that pair's offspring. Each offspring then has a probability *p* of undergoing *mutation*, or alteration of one randomly-selected beam to a new random value.

**Figure 1:**
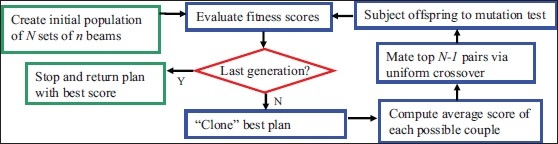
Flowchart of GA implementation. The algorithm proceeds for a set number of generations

The process is repeated with the new generation of *k* samples. After a number of generations, *s*, have been produced, the process is halted, and the sample with the best score is considered the solution to the BAS problem. In practice, the values of *k, p*, and *s* are selected based on the time and computational resources available, as discussed below. The GA process is illustrated in Figure 1.

### Evaluation

We analyzed an IMRT prostate case recently treated in our facility. The beam angles employed in the actual treatment were 45°, 105°, 180°, 255°, 315°. The DVH constraints for this case were those typically employed in our clinic. We used the GA algorithm described in Section A to produce each generation of beam-angle samples. The plans were generated with the Eclipse software running on four separate machines.

After each IMRT plan was generated, it was normalized so that 95% of the PTV received the prescription dose of 81Gy, and was then evaluated using the following scoring function:

S=ΣIΣjwi(Aj-Cj)

Where *A_j_* was the actual percentage volume receiving the constraint dose, *C_j_* was the percentage volume specified to receive the constraint dose, and *w_i_* was the relative weight assigned to the organ at risk (OAR). The sum over *i* included the bladder and rectum, and the sum over *j* included the constraints for each OAR. For this preliminary study, we used weights for the bladder and rectum of 1 and 2, respectively. Clearly, a lower score indicates a better treatment plan. The score for the clinical plan was -121.

We produced six generations of the GA, and in each case recorded the number of plans better than the clinical plan. We produced DVH figures to compare the best plan with the clinical plan.

## Results

**Figure 2:**
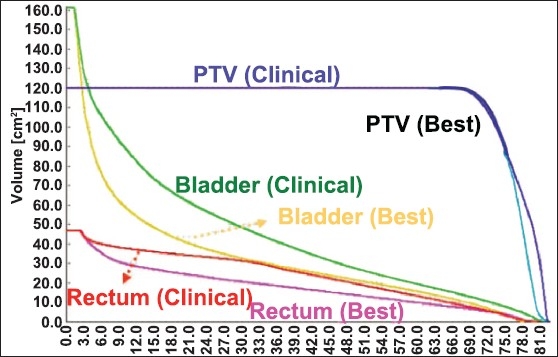
DVH comparison indicating the PTV, bladder, and rectum for the clinical and best GA plans

**Figure 3:**
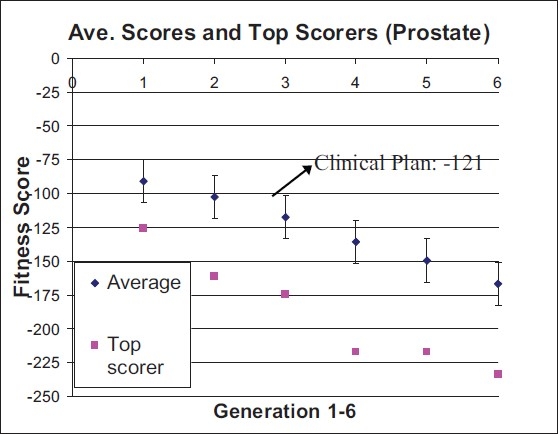
Average and best scores for each generation of the GA. Error bars represent standard deviations. Recall that lower numbers indicate better plans. The clinical plan is shown by a horizontal line for comparison. It can be seen that average and best plans improve as the GA proceeds

In Figure 2, we present the DVH's for the prostate, bladder, and rectum for the clinical and best GA plans. Clearly, the GA plan provides better sparing of the critical structures. Figure 3 presents, for each generation of samples, the average and best plan scores.

## Discussion and Conclusions

The GA is an effective method of searching the space of gantry angles. We have shown that it can produce IMRT prostate plans with significantly better fitness scores than typical clinical plans. The GA implementation for the BAS-DO problem gains power when IMRT plans corresponding to the beam-angle samples, for each iteration (see B1), are generated simultaneously. This requires distributing the samples over multiple IMRT workstations, or *computing nodes*, which is the subject of ongoing investigations. We have formed a collaboration with faculty members at the Center for Computational Research (CCR).[28,29] Access to the vast computational resources of the CCR will permit us to implement the GA algorithm to run simultaneously on *k* machines, with *k* in the range of 25-100 or more. The result is that the completion of each GA generation will require only approximately 30 minutes. This would enable 5-10 generations (*s* = 5-10) in the time currently required to produce one complete IMRT treatment plan.
